# Presepsin as a Novel Biomarker in Abdominal Sepsis: Diagnostic Accuracy and Prognostic Implications

**DOI:** 10.3390/biomedicines14040822

**Published:** 2026-04-03

**Authors:** Marco Fiore, Gianluigi Cosenza, Francesco Maria Romano, Vincenzo Pota, Pasquale Sansone, Francesco Coppolino, Lucio Selvaggi, Francesco Selvaggi, Maria Caterina Pace

**Affiliations:** 1 Department of Women, Child and General and Specialized Surgery, University of Campania “Luigi Vanvitelli”, 80138 Naples, Italy; vincenzo.pota@unicampania.it (V.P.); pasquale.sansone@unicampania.it (P.S.); francesco.coppolino@unicampania.it (F.C.); mariacaterina.pace@unicampania.it (M.C.P.); 2 Department of Advanced Medical and Surgical Sciences, University of Campania “Luigi Vanvitelli”, 80138 Naples, Italy; francescomaria.romano@unicampania.it (F.M.R.); lucio.selvaggi@unicampania.it (L.S.); francesco.selvaggi@unicampania.it (F.S.)

**Keywords:** presepsin, sCD14-ST, abdominal sepsis, intra-abdominal infection, sepsis biomarkers, postoperative infectious complications, SOFA score

## Abstract

**Background/Objectives:** Abdominal sepsis remains a major contributor to morbidity and mortality among surgical and critically ill patients worldwide. Timely diagnosis is frequently hindered by the overlapping clinical and biochemical features of postoperative inflammatory responses and evolving intra-abdominal infections, which may resemble systemic sepsis. Conventional biomarkers, including C-reactive protein (CRP) and procalcitonin (PCT), are widely implemented in clinical practice but demonstrate suboptimal specificity in differentiating infectious from sterile inflammatory conditions in the early postoperative phase. Presepsin (soluble CD14 subtype, sCD14-ST), a circulating fragment released during monocyte–macrophage activation in response to bacterial endotoxins, has emerged as a biomarker reflecting innate immune engagement. This review aims to critically evaluate the current evidence regarding the diagnostic accuracy, prognostic relevance, and potential clinical role of presepsin in abdominal sepsis. **Methods:** A comprehensive narrative review of the biomedical literature was performed using MEDLINE (via PubMed) and supplementary academic sources. Studies assessing the diagnostic performance, prognostic associations, and clinical applicability of presepsin in abdominal infections, postoperative infectious complications, and sepsis were systematically examined. Where available, comparative analyses with established biomarkers such as CRP and PCT were evaluated to contextualize its incremental value within existing diagnostic frameworks. **Results:** The accumulated evidence indicates that presepsin concentrations increase early during bacterial infections and correlate with validated severity indices, organ dysfunction scores, and mortality outcomes. Across multiple surgical and intensive care settings, presepsin demonstrated moderate-to-high diagnostic performance, frequently comparable to and occasionally exceeding that of traditional inflammatory biomarkers, particularly in distinguishing septic from non-septic inflammatory states. Moreover, dynamic changes in circulating levels appear to provide additional prognostic information and may support longitudinal clinical assessment. Nonetheless, substantial heterogeneity in study design, patient populations, sampling strategies, and reported cut-off values limits direct cross-study comparability and constrains definitive clinical recommendations. **Conclusions:** Presepsin represents a biologically plausible and clinically promising biomarker for the early identification and risk stratification of abdominal sepsis. Although current findings are encouraging, further large-scale, methodologically standardized prospective investigations are required to define optimal diagnostic thresholds and to clarify their role within multimodal biomarker strategies in contemporary sepsis management.

## 1. Introduction

Sepsis is currently defined as a life-threatening organ dysfunction resulting from a dysregulated host response to infection [1]. The Sepsis-3 consensus operationalizes organ dysfunction as an acute increase of at least two points in the Sequential Organ Failure Assessment (SOFA) score, reflecting clinically significant impairment across multiple physiological systems [2,3]. Despite advances in critical care management and antimicrobial stewardship, sepsis continues to represent a substantial global health burden. Among its diverse etiologies, intra-abdominal infections (IAIs) constitute a particularly relevant source in surgical populations, frequently leading to severe systemic complications and increased mortality [4,5].

Abdominal sepsis poses distinct diagnostic challenges. The clinical presentation is often heterogeneous and may overlap with postoperative physiological inflammatory responses, particularly in patients undergoing major abdominal procedures. Fever, leukocytosis, tachycardia, and elevated inflammatory markers may occur in both sterile postoperative inflammation and evolving infection, thereby complicating early recognition. Although prompt source control and timely antimicrobial therapy are well-established determinants of outcome, diagnostic uncertainty in the early phase remains a critical obstacle to optimal management.

Biomarkers are therefore widely employed to assist in clinical decision-making. C-reactive protein (CRP) and procalcitonin (PCT) are among the most commonly utilized inflammatory markers in surgical and intensive care settings. While both have demonstrated utility in identifying systemic inflammatory states, their specificity in differentiating bacterial infection from non-infectious inflammatory conditions remains suboptimal, particularly in the immediate postoperative context [6,7]. Consequently, there is sustained interest in identifying biomarkers that more directly reflect pathogen-driven immune activation.

Presepsin, also known as soluble CD14 subtype (sCD14-ST), has emerged as a candidate biomarker grounded in innate immune biology. CD14 is a glycoprotein expressed primarily on monocytes and macrophages and plays a central role in the recognition of lipopolysaccharide (LPS) and other pathogen-associated molecular patterns through Toll-like receptor-mediated signaling pathways [8,9,10]. During acute inflammatory activation, enzymatic cleavage of CD14 generates presepsin, a 13 kDa circulating fragment detectable in plasma through chemiluminescent immunoassays [10,11]. Because its release is mechanistically linked to microbial recognition rather than nonspecific inflammation, presepsin has been proposed as a biomarker with potentially greater specificity for bacterial infection compared with conventional inflammatory markers [12,13,14,15].

Over the past decade, an expanding body of literature has explored the diagnostic and prognostic implications of presepsin across various clinical contexts, including emergency medicine, critical care, and surgical populations. However, reported performance characteristics vary substantially across studies, and interpretation is complicated by differences in patient selection, timing of measurement, disease severity, and renal function status. In abdominal sepsis in particular, where diagnostic uncertainty frequently complicates early management, a critical appraisal of the available evidence is warranted.

The present narrative review aims to comprehensively evaluate the diagnostic accuracy, prognostic relevance, and potential clinical utility of presepsin in abdominal sepsis and related surgical settings, contextualizing current findings within the broader framework of contemporary sepsis management.

## 2. Materials and Methods

This study was conducted as a narrative review aimed at critically synthesizing the available evidence regarding the diagnostic and prognostic role of presepsin in abdominal sepsis and related surgical contexts. Given the heterogeneity of existing studies and the exploratory nature of the topic, a narrative approach was considered more appropriate than a formal systematic review or meta-analysis.

A comprehensive literature search was performed using the MEDLINE database (via PubMed) without temporal restriction up to the date of manuscript preparation. The search strategy incorporated combinations of the following keywords: “presepsin”, “sCD14-ST”, “abdominal sepsis”, “intra-abdominal infection”, “postoperative infectious complications”, and “sepsis biomarkers”. Boolean operators (AND, OR) were applied to refine search combinations and optimize retrieval. In addition, reference lists of relevant articles were manually screened to identify further pertinent studies not captured in the initial search.

Eligible publications included prospective and retrospective observational studies, randomized controlled trials, pilot studies, systematic reviews, and meta-analyses evaluating the diagnostic accuracy, prognostic value, or clinical applicability of presepsin in adult patients with abdominal infections, postoperative infectious complications, sepsis, or septic shock. Studies were included irrespective of clinical setting, encompassing surgical wards, intensive care units, and emergency departments. Only articles published in peer-reviewed journals and available in English were considered. Case reports, conference abstracts without full-text availability, and studies lacking a quantitative assessment of presepsin were excluded.

For each included study, relevant data were extracted, including year of publication, study design, sample size, patient population, sampling time points, reported diagnostic performance metrics (sensitivity, specificity, positive and negative predictive values, and, when available, area under the receiver operating characteristic curve), associations with severity scores (e.g., SOFA, APACHE II), and reported mortality outcomes. Where appropriate, comparisons with established biomarkers such as CRP, PCT, interleukin-6, or other inflammatory markers were documented to contextualize relative performance.

Given the methodological heterogeneity across studies—including differences in inclusion criteria, sepsis definitions (Sepsis-2 versus Sepsis-3), timing of measurement, and reported cut-off thresholds—a quantitative pooled analysis was not undertaken. Instead, findings were qualitatively synthesized and structured according to three principal domains: diagnostic discrimination, prognostic stratification, and longitudinal monitoring. This framework informed the organization of Table 1, Table 2 and Table 3 and guided the interpretative discussion.

No new patient data were generated or analyzed. Accordingly, institutional review board approval and informed consent were not required for the present study.

## 3. Results

The main findings of the included studies are summarized in Table 1 and Table 2. In accordance with the methodological framework adopted for this review, the available evidence is presented across three complementary domains: diagnostic discrimination, prognostic stratification, and longitudinal monitoring

### 3.1. Diagnostic Performance

Across heterogeneous surgical and critical care populations, presepsin demonstrated moderate-to-high diagnostic accuracy for identifying sepsis and septic shock. In postoperative abdominal surgery cohorts, early sampling frequently yielded clinically relevant discriminatory performance. In a study of 298 patients undergoing major abdominal procedures, sensitivity for sepsis reached 69.8% at baseline (T0) and increased to 76.9% at 24 h, while specificity reached 82% at 48 h. For septic shock, sensitivity was 83.8% at T0, with specificity up to 89.8% at 48 h [16]. In a prospective emergency surgical cohort, sensitivity and specificity were reported at 70% and 90%, respectively, with a positive predictive value of 90% [18].

Meta-analytic data further support diagnostic performance in the postoperative setting. In pooled analyses of postoperative infectious complications (*n* = 984), sensitivity and specificity were 76% and 83%, respectively. Similarly, in cirrhotic populations evaluated for bacterial infection, pooled sensitivity and specificity were 75% and 80% [29]. These findings indicate consistent discriminatory capacity across selected high-risk populations. In critically ill patients, performance characteristics varied according to disease severity and timing of measurement. In ICU cohorts, sensitivity values ranged from 79% [20] to 82–83% at 24–72 h [32], with specificity ranging from 63% [20] to 89.74% [28]. In febrile patients evaluated for early sepsis, sensitivity reached 89.5%, with a positive predictive value of 64.6% [30]. Conversely, in a large retrospective cohort of 2225 patients, specificity for septic shock reached 92.2%, although sensitivity was lower (39.6%) [35], reflecting stronger rule-in performance in advanced disease states.

Collectively, these data demonstrate that reported sensitivity typically ranges between approximately 60% and 90%, while specificity may reach values exceeding 90% in selected contexts (Table 2). However, variability in thresholds, sampling strategies, and comparator biomarkers contributes to inter-study heterogeneity.

### 3.2. Prognostic Associations

Beyond diagnostic discrimination, several studies reported significant associations between presepsin concentrations and indices of disease severity and mortality. Correlations with SOFA and APACHE II scores were consistently documented across multiple cohorts [20,21,26,35]. In the multicenter ALBIOS trial (*n* = 997), baseline concentrations increased proportionally with organ dysfunction severity, and early rises were associated with adverse clinical outcomes and increased 90-day mortality [21]. Similarly, observational data demonstrated higher baseline values in non-survivors compared with survivors [31], and a cut-off value exceeding 1.47 ng/mL was associated with mortality in septic shock patients [33]. In abdominal sepsis complicated by enterocutaneous fistula, concentrations greater than 726 pg/mL were associated with greater disease severity [26].

These findings indicate that baseline levels and early dynamic changes may reflect the intensity of systemic inflammatory activation and organ dysfunction burden. Nevertheless, heterogeneity in reported thresholds and outcome definitions limits direct comparability across studies.

### 3.3. Longitudinal Monitoring

A subset of studies evaluated serial measurements and their association with clinical course. In postoperative cohorts, persistent elevation during the early postoperative period was associated with infectious complications and organ dysfunction [22,25]. Measurements performed at postoperative days 5–7 demonstrated improved detection of infectious complications compared with conventional inflammatory markers [27]. In observational ICU studies, persistently elevated concentrations were associated with poorer prognosis, whereas decreasing trends were observed in patients demonstrating clinical improvement [32].

These observations suggest that temporal trajectories may provide additional contextual information beyond single baseline measurements. However, the optimal timing, frequency, and clinical integration of serial assessment remain insufficiently standardized.

## 4. Discussion

This narrative review synthesizes the current evidence regarding presepsin (soluble CD14 subtype, sCD14-ST) as a diagnostic, prognostic, and monitoring biomarker in abdominal sepsis. Collectively, the available data indicate that presepsin increases early during bacterial infection, correlates with organ dysfunction severity, and may provide clinically relevant prognostic information. Nonetheless, substantial heterogeneity in study design, patient populations, and diagnostic thresholds necessitates cautious interpretation.

From a mechanistic standpoint, presepsin reflects activation of the innate immune response through CD14-mediated recognition of pathogen-associated molecular patterns, particularly lipopolysaccharide derived from Gram-negative bacteria [8,9,10]. Proteolytic cleavage of CD14 during monocyte–macrophage activation generates circulating sCD14-ST, providing a biologically coherent explanation for its rapid elevation during systemic infection [10,11]. This mechanistic specificity distinguishes presepsin from nonspecific inflammatory markers such as CRP, which may increase in sterile inflammatory conditions [6,7], and supports its proposed relevance in differentiating infectious from non-infectious postoperative systemic inflammatory responses [12,13,14,15].

**Figure 1:**
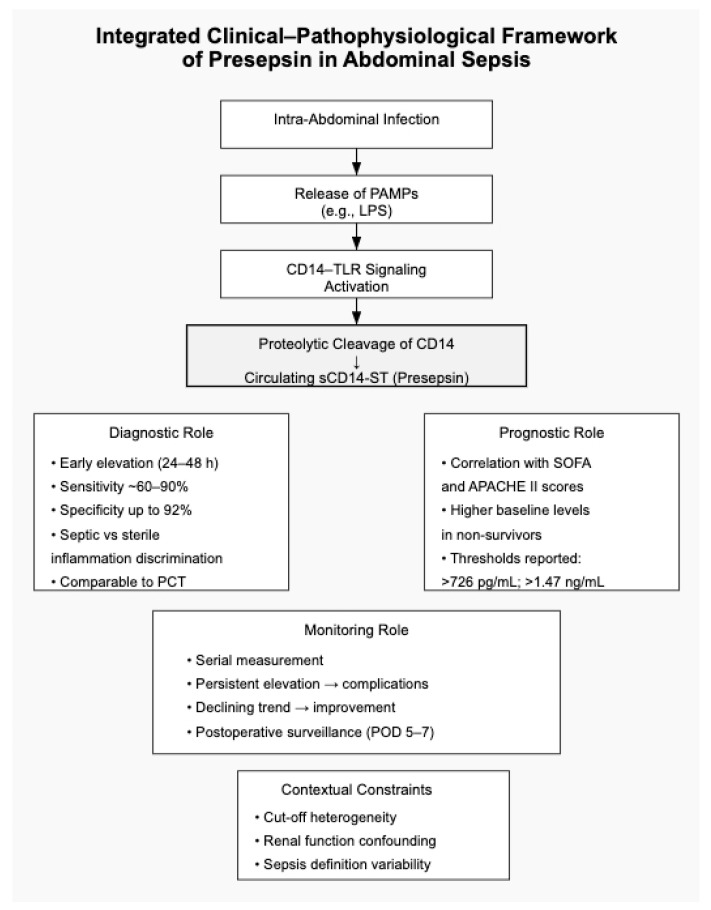
Pathophysiological and Clinical Integration of Presepsin in Abdominal Sepsis.

The cumulative evidence may be interpreted across three interrelated domains—diagnostic discrimination, prognostic stratification, and longitudinal monitoring—as summarized in Table 3. While Table 3 provides a structured comparative overview of these domains, the mechanistic and clinical interconnections underlying these roles are conceptually integrated in Figure 1. As illustrated, presepsin occupies a central interface between pathogen recognition and systemic inflammatory amplification. Its early elevation reflects innate immune activation; its magnitude correlates with organ dysfunction burden; and its temporal trajectory may offer additional contextual information regarding disease progression. Importantly, this integrated framework also highlights contextual modifiers—such as renal function and threshold variability—that influence interpretation and limit the applicability of universal cut-off values. As schematically represented, presepsin occupies a central interface between pathogen recognition and measurable systemic response. Its early rise reflects innate immune activation; its magnitude correlates with organ dysfunction burden; and its temporal trajectory may provide additional information regarding disease evolution. Importantly, the framework also emphasizes contextual modifiers, including renal function and disease severity, which influence circulating concentrations and must be considered during interpretation.

In surgical populations, particularly following major abdominal procedures, presepsin demonstrated moderate-to-high diagnostic accuracy [16,18,22,27]. Meta-analytic data reported pooled sensitivity and specificity of 76% and 83%, respectively, for postoperative infectious complications [19], while pooled estimates of 75% and 80% were observed in cirrhotic patients evaluated for bacterial infections [29]. These findings suggest clinically meaningful discriminatory capacity in high-risk settings characterized by diagnostic uncertainty.

Beyond diagnosis, several investigations consistently documented significant associations between presepsin concentrations and established severity indices, including SOFA and APACHE II scores [20,21,26,35]. In the ALBIOS cohort, early increases were associated with adverse outcomes [21], and higher baseline values were reported among non-survivors in independent cohorts [31,33]. These observations indicate that presepsin may reflect the intensity of systemic inflammatory activation and organ dysfunction, extending its relevance beyond binary diagnostic classification.

Nevertheless, comparative performance relative to established biomarkers remains heterogeneous. While certain studies reported comparable or superior diagnostic accuracy compared with procalcitonin [18,36], others observed stronger predictive value for bacteremia using PCT in selected contexts [37]. Such discrepancies likely reflect differences in infection source, sampling timing, disease severity, and applied cut-off thresholds.

Presepsin reaches higher concentrations in specific septic conditions, particularly in more severe forms of sepsis.

Its levels increase progressively with disease severity, being higher in severe sepsis and highest in septic shock. This makes presepsin a useful marker not only for diagnosis but also for assessing how advanced the condition is [38].

In addition, certain infection sites—such as respiratory, urinary, or biliary infections—may be associated with particularly elevated levels, especially when the infection is more severe or requires hospitalization [39].

Overall, presepsin correlates closely with clinical severity scores and prognosis: higher levels are associated with worse outcomes and increased risk of mortality.

Presepsin demonstrates distinct kinetic patterns between surgical contexts. In emergency abdominal surgery for intra-abdominal infection, presepsin elevates preoperatively (cutoff 350–407 pg/mL) and shows early postoperative predictive value [18,40]. Conversely, in elective colorectal surgery, presepsin remains relatively stable immediately postoperatively but increases on POD 4–6 when infectious complications develop, contrasting with CRP and PCT, which peak early and then decline [22]. This temporal difference makes presepsin particularly valuable for detecting delayed infectious complications after elective procedures [41].

Diagnostic accuracy in elderly patients (≥75 years) is comparable to that of the general population [28]. Importantly, presepsin levels change with age, but diagnostic thresholds do not require substantial age-based adjustment [8]. Combined use with PCT and early warning scores yields optimal diagnostic accuracy in very elderly emergency department patients [42]. 

Variability in reported diagnostic thresholds represents a major limitation. Cut-off values differ substantially across studies, ranging from lower thresholds in postoperative cohorts [26] to higher concentrations in septic shock populations [35]. Additionally, renal function significantly influences presepsin levels due to glomerular filtration and tubular metabolism [43,44]. Presepsin levels increase as renal function declines, showing a strong inverse relationship with eGFR. This makes interpretation challenging in patients with kidney disease, as elevated values may reflect reduced clearance rather than sepsis.

Levels rise progressively across CKD stages and can become very high in patients on hemodialysis, overlapping with those seen in severe sepsis. Therefore, standard cut-offs are not reliable: higher thresholds are needed in patients with reduced eGFR, although diagnostic accuracy decreases in advanced renal failure. 

Suggested thresholds are:

~500 pg/mL for eGFR ≥ 60 mL/min/1.73 m^2^

~1000 pg/mL for eGFR < 60 mL/min/1.73 m^2^

≥2200 pg/mL in severe renal impairment (creatinine > 4 mg/dL), though diagnostic accuracy decreases.

Overall, renal function must always be considered when interpreting presepsin, and in severe kidney impairment, alternative markers such as procalcitonin may be more reliable [45,46,47]. 

Presepsin levels decrease after effective antibiotic therapy, reflecting infection control and often changing earlier than other biomarkers.

A progressive decline is associated with good response and prognosis, while persistently high levels may indicate inadequate treatment or worse outcomes. Overall, presepsin is useful for monitoring therapy and guiding its duration [48].

Corticosteroids play an important role in the management of septic shock, where they help reduce mortality and modulate the excessive inflammatory response known as the cytokine storm. These drugs act by selectively reducing pro-inflammatory cytokines while preserving some immune function, and their combination with fludrocortisone may further improve clinical recovery [49,50,51].

Mesenchymal stem cells (MSCs) have shown promising effects in experimental models of sepsis. They appear to both enhance bacterial clearance and dampen excessive inflammation, mainly through paracrine mechanisms. MSCs can shift immune responses toward a more reparative profile, for example, by promoting anti-inflammatory macrophage activity. However, despite encouraging preclinical results and good safety data, their clinical use is still limited by inconsistent evidence and a lack of standardization [52,53].

Exosomes derived from MSCs represent a newer, cell-free therapeutic approach. They carry bioactive molecules such as microRNAs that regulate immune responses, reducing inflammation and promoting tissue protection. In some studies, they have shown even stronger anti-inflammatory effects than the cells themselves, although this field is still largely experimental [54].

As for presepsin, it is a biomarker that reflects activation of the innate immune system and correlates with sepsis severity. However, there is currently no direct evidence on how corticosteroids, MSCs, or exosome-based therapies affect its levels. Given their anti-inflammatory and immunomodulatory effects, it is plausible that these treatments could reduce presepsin concentrations, but this remains to be specifically demonstrated [55].

Presepsin levels are significantly higher in bacterial infections compared to viral ones, making it a useful marker for distinguishing bacterial from non-bacterial infections.

In viral infections, presepsin is usually lower, although it may increase in severe cases, such as advanced COVID-19, reflecting disease severity rather than bacterial involvement [40].

In fungal infections, presepsin levels are elevated and often comparable to those seen in bacterial infections. They also tend to correlate with disease severity, which can make differentiation between bacterial and fungal infections more challenging [56,57].

For this reason, combining presepsin with other biomarkers can improve diagnostic accuracy, especially in complex or immunocompromised patients.

Presepsin levels are generally somewhat higher in Gram-negative infections compared to Gram-positive ones, but the difference is modest and not consistent across all cases. Variability between individual bacterial species further limits the ability to clearly separate the two groups based on presepsin alone [37].

In clinical practice, these differences are not sufficient to reliably distinguish Gram-positive from Gram-negative infections. Nevertheless, presepsin remains a useful and reliable biomarker for the diagnosis of bacterial infections overall, regardless of the type of pathogen [58].

As highlighted in Figure 1, these contextual modifiers complicate the establishment of universally applicable thresholds and reinforce the need for population-specific interpretation.

In Table 4, comparative data between Presepsin and established core biomarkers (CRP and PCT) are shown [59].

Methodologically, most available studies are observational and vary in inclusion criteria, sampling schedules, and comparator biomarkers. Although cumulative evidence supports biological plausibility and clinical potential, the incremental contribution of presepsin within standardized multimodal diagnostic frameworks remains to be definitively established. Within the broader landscape of sepsis biomarker research, integrative approaches that combine pathophysiological specificity with contextual clinical interpretation are increasingly emphasized [1,60]. In this setting, presepsin appears most appropriately conceptualized as an adjunctive component within a composite diagnostic strategy rather than as a standalone determinant of clinical decision-making.

**Table 4:** Comparative data between Presepsin, PCT and CRP.

Characteristic	Presepsin	PCT	CRP References
**Biomarker Type**	sCD14-ST	Prohormone of calcitonin	Acute phase protein [59].
**Sensitivity for Sepsis**	82–84%	0.75–0.78 (75–78%)	Lower than presepsin/PCT [59].
**Specificity for Sepsis**	76–78%	0.75–0.77 (75–77%)	Lower than presepsin/PCT [37].
**AUROC for Sepsis Diagnosis**	0.87–0.88	0.84–0.86	0.85 [38].
**AUROC for Bacteremia**	0.79	0.86–0.88	0.55–0.60 [38].
**Diagnostic Odds Ratio**	16 (95% CI: 10–25)	Similar to presepsin	Inferior to presepsin/PCT [61].
**Positive Likelihood Ratio**	3.4 (95% CI: 2.5–4.6)	Similar to presepsin	Lower than presepsin/PCT [61].
**Negative Likelihood Ratio**	0.22 (95% CI: 0.17–0.27)	Similar to presepsin	Higher than presepsin/PCT [61].
**Prognostic Value (Mortality)**	Superior (AUROC 0.72, OR 3.31)	Moderate (AUROC 0.59, OR 1.62)	Inferior to presepsin/PCT [59].
**ICU Setting Sensitivity**	0.88 (95% CI: 0.82–0.92)	0.75 (95% CI: 0.68–0.81)	Not well-studied [59].
**ICU Setting Specificity**	0.58 (95% CI: 0.42–0.73)	0.75 (95% CI: 0.65–0.83)	Not well-studied [61].
**Combined Use Benefit**	Synergistic with PCT (AUROC 0.88)	Synergistic with presepsin	Limited added value [61].
**Pathogen Identification**	Associated with specific pathogens (*E. coli*, Klebsiella, Acinetobacter)	Associated with specific pathogens (*E. coli*, Klebsiella, Enterobacteriaceae)	Poor pathogen discrimination [59].
**Statistical Significance vs. PCT**	No significant difference (*p* = 0.169 sensitivity, *p* = 0.792 specificity)	Reference standard	Significantly inferior [37,59].
**Clinical Utility**	Better for prognosis and risk stratification	Better for bacteremia detection	Limited diagnostic value [37,61].

## 5. Conclusions

Presepsin is a biologically grounded biomarker reflecting innate immune activation during bacterial infection. The available evidence indicates moderate-to-high diagnostic performance in selected clinical contexts, meaningful correlation with organ dysfunction severity, and potential prognostic relevance in abdominal sepsis. However, variability in diagnostic thresholds, methodological heterogeneity, and renal function confounding limit the generalizability of current findings. Its clinical role appears most appropriate within a multimodal diagnostic framework, pending confirmation through standardized prospective investigations designed to clarify its incremental value over established biomarkers.

## Figures and Tables

**Table 1:** Main characteristics and findings of studies evaluating presepsin in abdominal sepsis and related settings.

Author (Ref.)	Year	Study Design	N	Sampling Time Points	Main Findings
Jeong et al. [16]	2022	Observational	298	T0, 24 h, 48 h, 72 h	Demonstrated diagnostic accuracy for sepsis and septic shock in the acute postoperative phase.
Paraskevas et al. [17]	2023	Systematic review	—	—	Identified as a promising biomarker for triage and early sepsis diagnosis.
Bösch et al. [18]	2020	Prospective	31	T0	Highest AUC, sensitivity, and specificity among evaluated markers; strong association with mortality.
Lu et al. [19]	2023	Meta-analysis	984	—	Pooled sensitivity 76% and specificity 83% for postoperative infectious complications.
Drăgonescu et al. [20]	2020	Prospective observational	114	T0	Higher levels in sepsis and septic shock; significant correlation with SOFA score.
Masson et al. [21]	2015	Multicenter randomized (ALBIOS)	997	T0, 24 h, 48 h, 7 d	Baseline concentrations increased with disease severity; early rise associated with worse outcomes and 90-day mortality.
Amanai et al. [22]	2022	Prospective observational	114	T0, 24 h, 48 h, 72 h, 4 d, 6 d	Postoperative increases at days 4–6 predicted infectious complications.
Ozdal et al. [23]	2024	Prospective	90	—	Elevated in appendicitis compared with controls; not discriminatory for complicated cases.
Sater et al. [24]	2025	Cross-sectional	129	T0	Strong correlation with septic shock parameters compared with other biomarkers.
Shakeyev et al. [25]	2022	Pilot study	36	T0, 72 h	Higher baseline levels associated with postoperative complications and organ dysfunction.
Song et al. [26]	2016	Prospective cohort	71	T0	Values > 726 pg/mL associated with greater disease severity.
Takeuchi et al. [27]	2020	Prospective cohort	30	T0, 24 h, 48 h, 72 h, 5 d, 7 d	Measurements at postoperative days 5–7 outperformed WBC, CRP, and PCT for infectious complications.
Wang et al. [28]	2020	Prospective	142	24 h, 72 h, 7 d	Elevated levels in septic elderly ICU patients; associated with 30-day mortality.
Wejnaruemarn et al. [29]	2025	Systematic review/meta-analysis	1789	—	Pooled sensitivity 75% and specificity 80% for bacterial infections.
Zong et al. [30]	2024	Prospective cohort	149	T0	Sensitivity 89.5% and PPV 64.6% for early sepsis detection.
Aliu-Bejta et al. [31]	2023	Observational	100	0–72 h	Higher baseline levels observed in non-survivors.
Chen et al. [32]	2020	Observational	60	0 h, 24 h, 4 d, 7 d	Persistent elevation associated with poorer prognosis.
Narendra et al. [33]	2022	Prospective cohort	92	T0, 24 h, 48 h, 72 h	Cut-off > 1.47 ng/mL predictive of mortality in septic shock.
Pluta et al. [34]	2024	Prospective	86	—	Correlated with positive blood cultures; not independently predictive of mortality.
Ren et al. [35]	2024	Retrospective	2225	T0, 24 h	Higher concentrations in SOFA > 5 and septic shock; high specificity (92.2%).

AUC, area under the curve; SOFA, Sequential Organ Failure Assessment; WBC, white blood cell count; CRP, C-reactive protein; PCT, procalcitonin; ICU, intensive care unit; PPV, positive predictive value; T0, baseline measurement.

**Table 2:** Diagnostic performance of presepsin in abdominal sepsis and related clinical settings.

Author (Ref.)	Clinical Setting	Sensitivity (%)	Specificity (%)	PPV (%)	NPV (%)	Comparator(s)
Jeong et al. [16]	Postoperative abdominal surgery	Sepsis: 69.8 (T0), 76.9 (24 h); Septic shock: 83.8 (T0)	Up to 89.8 (48 h)	—	—	PCT
Bösch et al. [18]	Emergency abdominal surgery	70	90	90	30	PCT, IL-6, WBC
Lu et al. [19]	Postoperative infectious complications (meta-analysis)	76	83	—	—	PCT, CRP
Drăgonescu et al. [20]	ICU patients	79	63	—	—	—
Amanai et al. [22]	Colorectal surgery	Up to 87.9 (72 h)	43.1–87.8 (time-dependent)	—	—	PCT, CRP, WBC
Wang et al. [28]	Elderly ICU patients	82.05–83.33 (24–72 h)	66.67–89.74	—	—	PCT, CRP, IL-6
Wejnaruemarn et al. [29]	Cirrhotic patients (meta-analysis)	75	80	—	—	PCT
Zong et al. [30]	Febrile patients	89.5	—	64.6	—	WBC, CRP, PCT
Chen et al. [32]	Sepsis monitoring	83	85	—	—	sTREM-1
Pluta et al. [34]	ICU sepsis	93	51	—	—	IL-6, PCT, CRP
Ren et al. [35]	Sepsis and septic shock	39.6	92.2	—	—	PCT, CRP

PPV, positive predictive value; NPV, negative predictive value; ICU, intensive care unit; PCT, procalcitonin; CRP, C-reactive protein; WBC, white blood cell count; IL-6, interleukin-6; sTREM-1, soluble triggering receptor expressed on myeloid cells-1; T0, baseline measurement.

**Table 3:** Comparative overview of the diagnostic, prognostic, and monitoring roles of presepsin in abdominal sepsis and related settings.

Clinical Role	Key Evidence (Refs.)	Main Findings	Clinical Implication
**Diagnostic**	[16,18,19,20,22,27,28,29,30,35]	Sensitivity generally ranges from 60 to 90%; specificity up to 92.2% in septic shock; pooled sensitivity 76% and specificity 83% for postoperative infectious complications; pooled sensitivity 75% and specificity 80% in cirrhotic infections.	May provide adjunctive diagnostic information in selected clinical settings, particularly when interpreted alongside established biomarkers and clinical assessment.
**Prognostic**	[21,26,31,33,35]	Baseline concentrations correlate with SOFA score and disease severity; early increases associated with worse outcomes; cut-off > 1.47 ng/mL predictive of mortality; values > 726 pg/mL associated with greater clinical severity.	May contribute to risk stratification; however, prognostic performance appears context-dependent and requires validation in standardized prospective cohorts.
**Monitoring**	[22,25,27,32]	Persistent postoperative elevation associated with infectious complications and organ dysfunction; measurements at postoperative days 5–7 improved detection of complications; decreasing trends associated with clinical improvement.	Serial measurements may support clinical monitoring in postoperative and critical care contexts, although optimal timing and thresholds remain to be standardized.

SOFA, Sequential Organ Failure Assessment; ng/mL, nanograms per milliliter; pg/mL, picograms per milliliter.

## Data Availability

This manuscript constitutes a narrative review of previously published literature. No new data were generated or analyzed during the study; consequently, data availability is not applicable.

## Abbreviations

The following abbreviations are used in this manuscript:

ALBIOS	Albumin Italian Outcome Sepsis trial
APACHE II	Acute Physiology and Chronic Health Evaluation II
AUC	Area under the curve
cIAIs	Complicated intra-abdominal infections
CRP	C-reactive protein
ECF	Enterocutaneous fistula
ED	Emergency department
GFR	Glomerular filtration rate
IAI	Intra-abdominal infection
ICU	Intensive care unit
IL-6	Interleukin-6
IL-8	Interleukin-8
INR	International normalized ratio
LPS	Lipopolysaccharide
MAP	Mean arterial pressure
NPV	Negative predictive value
OD	Organ dysfunction
PAMPs	Pathogen-associated molecular patterns
PCT	Procalcitonin
PIC	Postoperative infectious complications
PLT	Platelet count
PPV	Positive predictive value
PSP	Presepsin
ROC	Receiver operating characteristic
SAP	Severe acute pancreatitis
SBP	Spontaneous bacterial peritonitis
SIRS	Systemic inflammatory response syndrome
SOFA	Sequential Organ Failure Assessment
sCD14-ST	Soluble CD14 subtype
sTREM-1	Soluble triggering receptor expressed on myeloid cells-1
TLRs	Toll-like receptors
uPAR	Urokinase-type plasminogen activator receptor
WBC	White blood cell count
WSES	World Society of Emergency Surgery
